# In Memorian Thomas W. Hesterberg, PhD MBA (1950 – 2016)

**DOI:** 10.1186/s12989-017-0188-2

**Published:** 2017-03-22

**Authors:** David B. Warheit

**Affiliations:** Wilmington, Delaware USA

Tom Hesterberg, a great friend and colleague, suffered an untimely death in August, 2016. I met Tom in the first half of 1981. I had recently arrived in the Fall of 1980 at the National Institute of Environmental Health Sciences (NIEHS) as a postdoctoral fellow. The NIEHS Branch Chief of the Laboratory of Pulmonary Function and Toxicology at that time, Dr. Paul Nettesheim, had arranged for Tom and I to have dinner at Darryl’s restaurant in Chapel Hill, NC to discuss a funding pathway to bring Tom to NIEHS. I remember our first meeting almost like it occurred yesterday. From the outset, I was very impressed with Tom’s friendliness, intelligence, and convivial personality. Working with Dr. Carl Barrett, Tom wrote and submitted an NIH grant application, designed to investigate potential mechanisms of asbestos-related carcinogenicity, and subsequently was awarded the highest priority score in that NIH postdoctoral grant cycle. It was important that Tom, in receiving this extramural NIH grant, achieve success in bringing his own funds to NIEHS. Tom also had great success in his years at NIEHS and published several important papers, (in Cancer Research, Carcinogenesis, and other journals) related to mechanisms of asbestos-mediated carcinogenesis. I believe that Tom was a major contributor in launching Carl Barrett’s Laboratory into the forefront of fibre carcinogenesis research.

**ᅟ:**
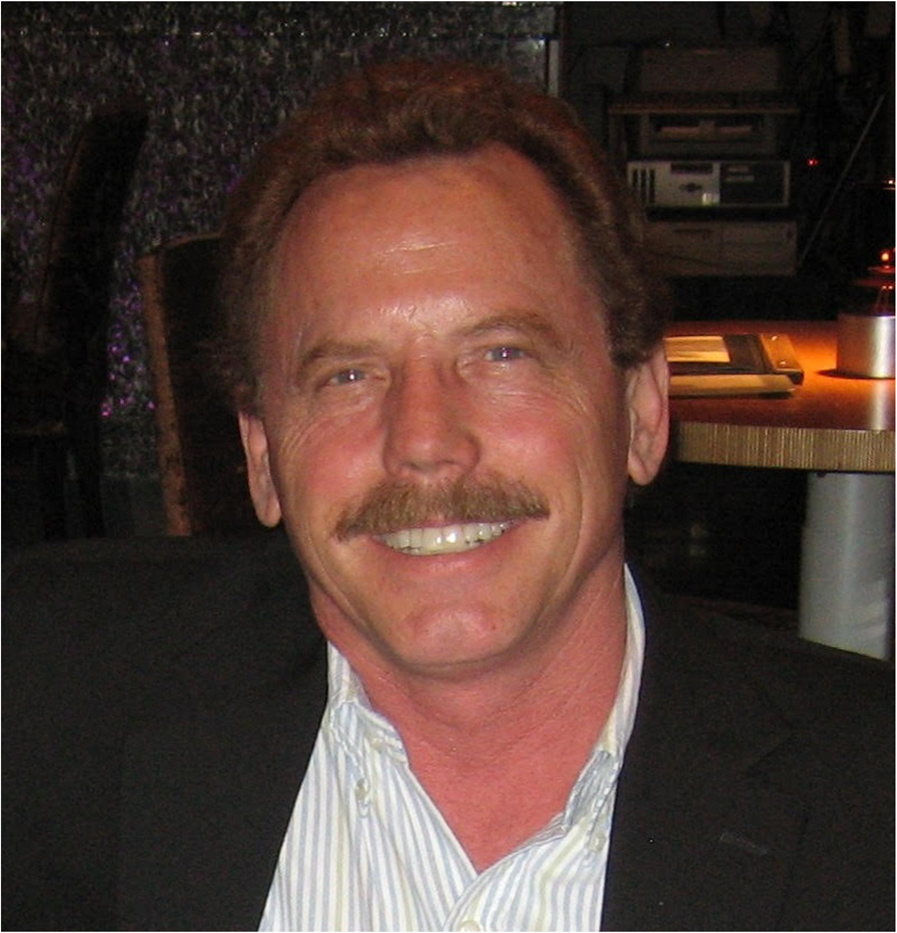
Thomas W. Hesterberg

Following his successful experience at NIEHS, Tom accepted a position as visiting scientist in the Genetic Toxicology Department at the Chemical Industry Institute of Toxicology (CIIT). During his tenure at CIIT, Tom developed a human bronchial epithelial cell culture system to be utilized for in vitro carcinogenesis studies. A number of publications in the journal Carcinogenesis are reflective of his scientific productivity while at CIIT.

Based upon his research experiences at NIEHS and CIIT, Tom was well positioned to join the Johns Manville Company during a critical period when the company and industry in general were contemplating conducting fibre toxicity research with a variety of man-made vitreous fibre-types. Without a doubt, Tom led the international industry effort to assess the safety of these synthetic inorganic fibre-types in what became known as the ‘RCC studies’. In this regard, he was the scientist primarily responsible for designing the subchronic and chronic inhalation studies. He took the lead as a study monitor to ensure that the studies were conducted in a competent and exemplary fashion. In addition, he developed and standardized methodologies for counting and sizing fibres retained from the lungs of exposed animals and innovated new in vitro systems for assessing the likelihood of biopersistence of synthetic fibre-types in vivo. Tom published and presented both the in vivo and in vitro study results in numerous journals and international forums and these had a profound influence on regulation and indeed set the standard for the future conduct of inhalation exposure studies with fibres. Tom was widely recognized for his expertise in fibre toxicology. Tom was an effective proponent of the structure/toxicity relationship that emerged from the totality of the RCC studies and that has dominated fibre toxicology ever since. This concept is that inhaled fibres of low durability or biopersistence produced significantly less hazard or risk to workers/consumers when compared with inhalation exposures to durable or biopersistent fibres such as asbestos or other forms of durable man-made fibre-types.

A major impact of Tom’s research efforts in the RCC studies was the 2001 decision of an International Agency for Research on Cancer (IARC) panel to classify biosoluble man-made vitreous fibres as “not classifiable to human carcinogenicity”. Based on his extensive published research findings in the field, Tom was selected by IARC to serve on the review panel. That was in itself a noteworthy achievement recognizing that scientists employed in industry are not often requested to serve on IARC panels.

Following his experience with man-made mineral fibres, Tom spent 10 years at Navistar, Inc. the largest manufacturer of trucks and engines in the United States, where he served as Director of Product Stewardship, Sustainability and Environmental Health. At Navistar, he oversaw the evaluation of the potential health, safety and environmental risks related to the use of diesel trucks and engines. Following his tenure at Navistar, Tom served as a Principal Toxicologist at the Center for Toxicology and Environmental Health.

On a more personal note, Tom’s curriculum vitae and bibliography were very impressive. He published over 100 scientific papers and was a recognized expert in fibre and particle toxicology, serving on numerous expert scientific panels. Tom’s efforts and accomplishments provided a significant positive impact on the field of toxicology and were highly regarded by scientific regulators, both in the US, Europe and Australia as well as with academic and industrial colleagues. In 2006, Tom was awarded the prestigious Career Achievement Award by the Society of Toxicology, Inhalation Specialty Section. He served on several editorial boards of leading toxicology journals and numerous national and international scientific panels. Tom was very bright, gregarious and engaging, with a great smile. He was an intellectually curious individual who was highly motivated, friendly, with diverse, eclectic interests; as well as a life-long learner, evidenced by the fact that he had attained an MBA while working as a Toxicology Director at the Johns Manville Company.

I will miss my good friend Tom Hesterberg, and he will be missed by the entire particle and fibre toxicology community.

