# Erratum to: In Memorian Thomas W. Hesterberg, PhD MBA (1950–2016)

**DOI:** 10.1186/s12989-017-0195-3

**Published:** 2017-04-27

**Authors:** David B. Warheit

**Affiliations:** Wilmington, Delaware USA

## Erratum

After the publication of this work [1] it was noticed that the title of the article was incorrect. The correct title should read: Tom Hesterberg—In Memoriam.
